# HLA-matched sibling transplantation with G-CSF mobilized PBSCs and BM decreases GVHD in adult patients with severe aplastic anemia

**DOI:** 10.1186/1756-8722-3-51

**Published:** 2010-12-31

**Authors:** Sun Zi-Min, Liu Hui-Lan, Geng Liang-Quan, Wang Xin-Bing, Yao Wen, Liu Xin, Ding Kai-Yang, Han Yong-Sheng, Yang Hui-Zhi, Tang Bo-lin, Tong Juan, Zhu Wei-Bo, Wang Zu-Yi

**Affiliations:** 1 Department of Hematology, Anhui Medical University Affiliated Anhui Provincial Hospital, Hefei, China

## Abstract

**Background:**

Allogeneic hematopoietic stem cell transplantation (allo-HSCT) is an effective treatment for severe aplastic anemia (SAA). However, graft failure and graft-versus-host disease (GVHD) are major causes of the early morbidity in Allo-HSCT.

**Methods:**

To reduce graft failure and GVHD, we treated fifteen patients with SAA using high- dose of HSCT with both G-CSF mobilized PB and BMSCs from HLA-identical siblings to treat patients with SAA.

**Results:**

All patients had successful bone marrow engraftment. Only one patient had late rejection. Median time to ANC greater than 0.5 × 10^9^/L and platelet counts greater than 20 × 10^9^/L was 12 and 16.5 days, respectively. No acute GVHD was observed. The incidence of chronic GVHD was 6.67%. The total three-year probability of disease-free survival was 79.8%.

**Conclusion:**

HSCT with both G-CSF mobilized PB and BMSCs is a promising approach for heavily transfused and/or allo-immunized patients with SAA.

## To the Editor

Allogeneic hematopoietic stem cell transplantation (allo-HSCT) is a cure for patients with severe aplastic anemia (SAA). However, complications such as graft failure and graft-versus-host disease (GVHD) have limited the application of allo-HSCT[1,2]. Increasing the number of donor blood stem cells decreases graft failure, however, high-dose of blood stem cell transplantation also increases the incidence of GVHD[3]. A combination of un-manipulated marrow and T-cell depleted PBSC as the stem cell source for SAA have shown rapid engraftment without increasing the risk of GVHD [4,5]. Here, we report that transplantation of un-manipulated peripheral blood stem cells (PBSC) combined with bone marrow stem cells (BMSC) in patients with SAA to reduce the incidence of graft failure without negative effects on GVHD.

Fifteen SAA patients, received HLA- 6/6-identical sibling G-CSF-mobilized PB plus BMSC transplantation (Table 1). CY/ALG (12/15 patients) or Flu/CY/ALG (3/15 patients) were used as conditioning regimen for all of them. CsA-MMF regimen was used to prevent aGVHD. Other supportive treatment were also given, such as *a*cyclovir, intravenous rhG-CSF, and intravenous immunoglobulin. The engraftment of transplant cells was determined using the following methods: STR-PCR analysis, Y PCR analysis, and tests for hematopoietic reconstitution and GVHD.

**Table 1:** Outcome of 15 SAA patients who received the PB+BM transplantation

No.	Disease	Conditioning Regimen	GVHD Prophylaxis	Cell number		Engraftment (days)						
				NC × 10^8^/kg	CD34 × 10^6^/kg	ANC	PLt					
				PB/BM	PB/BM	> 0.5 × 10^9^/L	> 20 × 10^9^/L	> 50 × 10^9^/L	Acute GVHD	chronic GVHD	Survival (Month)	Cause of death
1	VSAA-I	CY/ALG	CsA+MMF	5.95/3.06	3.07/0.89	11	15	18	N	skin	80^+^	
2	VSAA-I	CY/ALG	CsA+MMF	2.47/1.9	2.39/0.7	11	14	18	N	N	62^+^	
3	SAA-II	CY/ALG	CsA+MMF	2.91/2.6	2.33/1.48	15	47	53			7	Late graft
									N	N		Rejection
4	VSAA-I	CY/ALG	CsA+MMF	2.46/2.21	5.66/0.95	14	22	34	N	N	54^+^	
5	SAA-I	CY/ALG	CsA+MMF	6.47/1.88	5.3/0.47	10	20	50	N	N	9	Infection
6	SAA-I	CY/ALG	CsA+MMF	4.54/3.87	2.81/1.1	12	20	32	N	N	46^+^	
7	VSAA-I	CY/ALG	CsA+MMF	6.17/1.0	1.54/0.3	14	30	35	N	N	30^+^	
8	SAA-I	CY/ALG	CsA+MMF	4.64/1.86	4.45/0.71	11	15	18	N	N	30^+^	
9	SAA-II	Flu/CY/ALG	CsA+MMF	5.05/1.14	1.36/0.33	12	17	20	N	N	29^+^	
10	SAA-II	Flu/CY/ALG	CsA+MMF	3.75/1.47	4.2/0.66	12	15	16	N	N	28^+^	
11	SAA-I	CY/ALG	CsA+MMF	2.98/1.77	6.62/0.9	10	15	20	N	N	26^+^	
12	VSAA-I	CY/ALG	CsA+MMF	7.80/2.6	5.7/0.85	12	14	15	N	N	26^+^	
13	SAA-II	Flu/CY/ALG	CsA+MMF	5.86/2.1	5.03/0.92	13	16	16	N	N	20^+^	
14	VSAA-I	CY/ALG	CsA+MMF	2.15/1.9	0.49/1.14	23	27	35	N	N	5	Infection
15	SAA-I	CY/ALG	CsA+MMF	8.3/0.77	1.66/0.17	16	29	48	N	N	7^+^	
Median (range)				4.64(2.15-8.3)/1.9(0.77-3.87) × 10^8^/kg	3.07(0.49-6.62)/0.85(0.17-1.48) × 106/kg	Day 12 (10-23)	Day 16.5 (14-47)	Day 20 (15-53)			Month 27 (7-80)	

CY: cyclophosphamide; ALG: antihuman T-lymphocyte globulin; MMF: mycophenolate mofetil; CsA: cyclosporine A; N: without any acute GVHD or chronic GVHD

**Figure 1:**
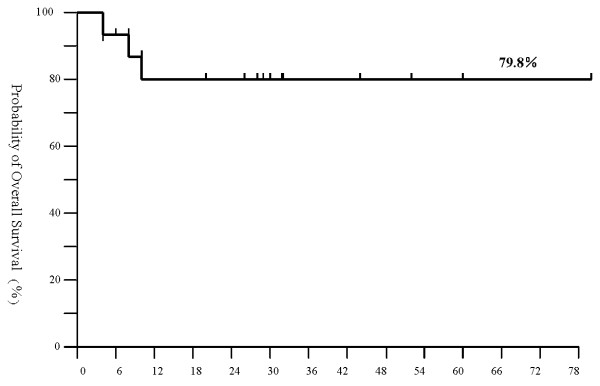
**Kaplan-Meier estimates overall survival rate of SAA patients treated with CsA and MMF after bone marrow and peripheral blood stem cell translantation from HLA-matched donors**.

All fifteen patients receiving allo-HSCT had successful bone marrow engraftment except for one of them had a late rejection. The incidence of acute and chronic GVHD was 0% and 6.67%. The reasons for the decreased incidence may be multifactorial, the use of G-CSF mobilized PBSC + BMSC_S _as the source of grafts, usage of ALG in conditioning regimen and CsA/MMF for the prophylaxis of GVHD. No recipients died from treatment-related complications within the first 100 days after transplantation. There were twelve disease-free survivals. The total three-year probability of disease-free survival was 79.8% (Figure 1).

Our data indicate that high- dose of HSCT with both G-CSF mobilized PB and BMSCs resulted in a quick engraftment, low graft rejection, a relatively low incidence of acute GVHD, and good DFS, although larger scale, prospective, and randomized studies are required to confirm these benefits.

## List of abbreviations

allo-HSCT: Allogeneic hematopoietic stem cell transplantation; SAA: severe aplastic anemia; GVHD: graft-versus-host disease; ANC: absolute neutrophil count; MSCs: mesenchymal stem cells; MPCs: mesenchymal (stroma) progenitor cells.

## Competing interests

The authors declare that they have no competing interests.

## Authors' contributions

SZM have made substantial contributions to conception and design; LHL, GLQ and WXB participated in the acquisition of data; WZY and ZWB participated in analysis and interpretation of data; YW and TJ drafted the manuscript; HYS, YHZ and LX revising it critically for important intellectual content; DKY and TBL performed the statistical analysis.

All authors have read and approved the final manuscript.
